# Insular Involvement in Cases of Epilepsy Surgery Failure

**DOI:** 10.3390/brainsci12020125

**Published:** 2022-01-18

**Authors:** Jimmy Li, Sandra Reiter-Campeau, Dina Namiranian, Dènahin Hinnoutondji Toffa, Alain Bouthillier, François Dubeau, Dang Khoa Nguyen

**Affiliations:** 1 University of Montreal Health Center Research Center (CRCHUM), 900 St.-Denis Street, Montreal, QC H2X 0A9, Canada; denahin.hinnoutondji.toffa@umontreal.ca (D.H.T.); d.nguyen@umontreal.ca (D.K.N.); 2 Neurology Division, University of Sherbrooke Health Center (CHUS), Sherbrooke, QC J1H 5H3, Canada; 3 Montreal Neurological Institute and Hospital (MNI/MNH), Montreal, QC H3A 2B4, Canada; sandra.reiter-campeau@mail.mcgill.ca (S.R.-C.); francois.dubeau@mcgill.ca (F.D.); 4 Neurology Division, Jewish General Hospital (JGH), Montreal, QC H3T 1E2, Canada; dina.namiranian@mcgill.ca; 5 Neurosurgery Division, University of Montreal Health Center (CHUM), Montreal, QC H2X 3A4, Canada; alain.bouthillier@umontreal.ca; 6 Neurology Division, University of Montreal Health Center (CHUM), Montreal, QC H2X 3A4, Canada

**Keywords:** epilepsy surgery, surgery failure, insular epilepsy, temporal-plus, operculo-insular, insula, epilepsy

## Abstract

Background: Epilepsy surgery failure is not uncommon, with several explanations having been proposed. In this series, we detail cases of epilepsy surgery failure subsequently attributed to insular involvement. Methods: We retrospectively identified patients investigated at the epilepsy monitoring units of two Canadian tertiary care centers (2004–2020). Included patients were adults who had undergone epilepsy surgeries with recurrence of seizures post-operatively and who were subsequently determined to have an insular epileptogenic focus. Clinical, electrophysiological, neuroimaging, and surgical data were synthesized. Results: We present 14 patients who demonstrated insular epileptic activity post-surgery-failure as detected by intracranial EEG, MEG, or seizure improvement after insular resection. Seven patients had manifestations evoking possible insular involvement prior to their first surgery. Most patients (8/14) had initial surgeries targeting the temporal lobe. Seizure recurrence ranged from the immediate post-operative period to one year. The main modality used to determine insular involvement was MEG (8/14). Nine patients underwent re-operations that included insular resection; seven achieved a favorable post-operative outcome (Engel I or II). Conclusions: Our series suggests that lowering the threshold for suspecting insular epilepsy may be necessary to improve epilepsy surgery outcomes. Detecting insular epilepsy post-surgery-failure may allow for re-operations which may lead to good outcomes.

## 1. Introduction

Epilepsy surgery failure occurs in approximately 20–30% of temporal lobe surgeries and 30–50% of frontal lobe surgeries [1,2]. Several hypotheses have been proposed to explain this elevated rate of epilepsy surgery failure. In temporal lobe epilepsy (TLE) surgery, which remains the most common surgical intervention for drug-resistant epilepsy, explanations that are classically put forward for failures include insufficient resection of epileptogenic tissue, dual pathology (the coexistence of mesial temporal sclerosis (MTS) and a neocortical lesion), contralateral mesial TLE relapse, extra-temporal lobe epilepsy mimicking TLE, and temporal-plus epilepsy, namely, a multilobar epilepsy with a primary temporal epileptogenic zone involving adjacent regions [3].

The insular lobe, a structure extensively connected with surrounding lobes, is increasingly recognized as being a “great mimicker” of other forms of focal epilepsy and participates in widespread epileptogenic connections. The lack of recognition of insular epilepsy could explain some temporal and extratemporal epilepsy surgery failures [4]. The challenge of recognizing insular involvement in the genesis of focal seizures lies in the fact that its semiology can mimic frontal, temporal, or parietal lobe seizures, and that insular epilepsy is often not “pure”, instead extending to adjacent opercula or coexisting with lobar epilepsies (i.e., insular-plus epilepsy) [5].

In addition, the contribution of most non-invasive investigations in detecting insular epilepsy is limited. Magnetic resonance imaging (MRI) may be useful in the visualization of an insular lesion; however, insular epilepsies are commonly non-lesional. Scalp electroencephalography (EEG) is unable to distinguish spikes originating from the deep-seated insula versus the overlying cortices [6]. Single-photon emission computed tomography (SPECT) and positron emission tomography (PET) may reveal changes in the insular lobe, but these techniques’ sensitivity and specificity remain limited due to their spatial and temporal resolution [5]. The use of magnetoencephalography (MEG), which measures magnetic fields produced by epileptic discharges, has been found to be a valuable non-invasive tool in identifying insular sources [7,8,9,10,11,12]. MEG has also been suggested to carry advantages for localization in patients with previous surgeries since MEG signals are less altered by skull defects or surgical cavities as compared to EEG signals [13]. Nevertheless, intracranial EEG (icEEG) with adequate sampling of the insula using intracerebral depth electrodes provides the best approach to confirm an insular focus in most patients with a suspicious clinical presentation [14].

The objective of this retrospective series is to review the clinical, electrophysiological, neuroimaging, and surgical characteristics of cases of epilepsy surgery failure subsequently attributed to an insular focus. We report 14 such cases and aim to identify shared patterns from these cases to help better guide clinical care.

## 2. Materials and Methods

We retrospectively identified adult patients investigated between 2004 and 2020 at the epilepsy monitoring units of the Centre Hospitalier de l’Université de Montréal and of the Montreal Neurological Institute and Hospital (Montreal, QC, Canada). Included patients met the following criteria: (a) had undergone one or multiple epilepsy surgeries with recurrence of seizures post-operatively; and (b) subsequently determined to have either a definite or probable insular/insular-plus epileptogenic focus. No follow-up duration threshold was set for evaluating seizure recurrence (i.e., seizure recurrence could be noted in the immediate post-operative period just as it could be noted years after the surgery). Definite insular involvement was confirmed by icEEG (by demonstration of seizures arising from the insula) or seizure control after insular lobe surgery, and probable insular involvement was based on MEG results revealing a cluster of sources in the insula (i.e., ≥6 spike sources with ≤1 cm between each other). If a patient fulfilled criteria for both definite and probable insular involvement (e.g., a patient had positive MEG and icEEG findings post-surgery-failure), this patient was considered as having definite insular involvement.

Data retrieved from pre-surgical/peri-surgical evaluations included the following: gender, age at seizure onset, presence of epilepsy risk factors, seizure semiology, preoperative investigation findings, including results from scalp EEG, video-EEG, brain MRI, SPECT, cerebral PET scan, MEG, and icEEG, surgical interventions performed, and pathology findings. The scope of the initial failed epilepsy surgery’s presurgical evaluation was heterogeneous, changing over time and depending on the institution performing the surgery. Notably, in our centers in Montreal, MEG was only available starting in 2006 and was initially used purely for research purposes, performed only selectively in earlier years as we were becoming familiar with this new technique. Data retrieved from post-surgical evaluations included time to seizure recurrence, semiology change after the first intervention, as well as any other investigations performed at the discretion of the treating physician. All but one patient underwent an MEG after their initial surgery failure as part of their work-up. Data on clinical outcome following the last surgical intervention were also gathered.

## 3. Results

We present 14 patients who were found to have an insular (or insular-plus) focus contributing to their epilepsy as detected by icEEG, MEG, or seizure improvement after insular resection. Based on our defined criteria, eight patients were found to have probable insular involvement, and six were found to have definite insular involvement. Table 1 consists of a summary description of our cohort. Table 2 details the patients’ baseline, presurgical, and peri-surgical characteristics. Table 3 details their clinical and paraclinical characteristics following failed epilepsy surgery and provides data on any further interventions and follow-up.

### 3.1. Patient and Seizure Characteristics

Eleven patients were men, and four had identifiable seizure risk factors. The mean age at seizure onset was 16 years (range 2–40 years). Two patients had only focal aware seizures, whereas the other 12 patients had focal seizures with impaired awareness; eleven had had a focal-to-bilateral tonico-clonic (FBTC) seizure at some point. The majority presented predominantly sleep-related seizures (10/14).

In descending order of frequency, the most frequent symptoms or manifestations were as follows: somatosensory symptoms (6/14), oro-alimentary automatisms (6/14), epigastric sensations (5/14), hyperkinetic seizures (4/14), clonic motor manifestations (4/14), and auditory illusions (4/14). Less frequent semiological features included dystonic posturing (3/14), olfacto-gustatory symptoms (3/14), déjà-vu (3/14), autonomic symptoms (3/14), emotional manifestations (3/14), hypersalivation (2/14), language disturbances (2/14), behavioral arrest (2/14), throat constriction/dysesthesias (2/14), upper extremity automatisms (2/14), and gelastic seizures (1/14). Among the six patients with somatosensory manifestations, two had ipsilateral symptoms, two had contralateral symptoms, one had bilateral appendicular symptoms, and one had truncal symptoms. These manifestations were described as painful in two patients (patients #8 and #12) and uncomfortable in one patient (patient #14). Six patients presented various combinations of auras (e.g., patient #1 with early viscerosensory, visceromotor, and somatosensory symptoms).

### 3.2. Pre-Surgery-Failure Neurophysiology and Neuroimaging Data, and Failed Surgical Intervention

Presurgical scalp EEG data was available for all patients. Interictally, nine EEGs suggested a temporal focus, three a frontal focus, and two a fronto-temporal focus. Ictally, seven EEGs suggested a temporal focus, one a frontal focus, and one a fronto-temporal focus; the remaining five ictal EEGs were non-localizing. Eight of the 14 patients underwent icEEG, the majority without any insular coverage; the insula was sampled using a single depth electrode in only three patients. In these three cases, the depth electrode was placed freehandedly after open craniotomy and opening of the Sylvian fissure [15]. The insular depth electrode placement was not optimal; for patients #2 and #3 the electrode was placed in the posterior insula, while for patient #8 the electrode did not reach the insula. In the first patient (#2) with insular sampling, recordings revealed epileptiform discharges from the frontal and temporal opercula but also from the medial frontal gyrus and adjacent mid-cingulate cortex. In the second patient (#3), the onset appeared to be from the right frontopolar region with propagation to the anterior cingulate cortex and operculo-insular region. In the third patient (#8), icEEG recorded discharges originating from the left hippocampus and Heschl’s gyri. In the five remaining patients without insular sampling, icEEG suggested frontal seizure onset zones in two patients and temporal zones in three patients; icEEG was non-localizing in one patient.

Twelve presurgical MRI reports were available: five patients had mesial temporal sclerosis (MTS), three had unremarkable MRIs, and two had temporal lobe atrophy without MTS. The two remaining patients had abnormalities involving the insula on MRI: patient #8 showed a focal cortical dysplasia at the junction of the left posterior insula and Heschl’s gyrus, and patient #12 had subtle thickening of the left insular cortex, which was initially interpreted to be of uncertain significance.

Eight patients had presurgical PET scans, none suggesting an insular focus. Hypometabolism of the mesiotemporal structures was found in five patients. In three of these patients, hypometabolism extended to adjacent regions (frontopolar, lateral temporal, and lateral and polar temporal). A sixth patient also presented an expected mesiotemporal hypometabolism following a first failed selective amygdalohippocampectomy (SAH). One patient had hypometabolism of the frontal lobe, and another had post-ictal hypermetabolism in the lateral temporal cortex.

Nine patients underwent a SPECT study, with 14 ictal SPECTs in seven patients and two interictal in two other patients. Only one ictal SPECT localized activation to the operculo-insular region (patient #2); the remaining ictal SPECTs showed activation in various temporal, frontal, or fronto-temporal regions.

Four patients underwent presurgical MEG: one MEG localized to the insula, and the other three localized to extra-insular regions. Patient #1 presented (in a MEG done after a selective right amygdalohippocampectomy) sources in the right anterior insula, orbitofrontal cortex, and non-resected anterior temporal region, patient #3 showed contralateral mesiofrontal sources, patient #6 had a cluster of sources at the junction of the right anterior insula, right posterior orbitofrontal cortex, and right frontal operculum, and, finally, left anterior temporal dipoles were identified in patient #13.

Eight patients had initial epilepsy surgeries targeting the temporal lobe, five had surgeries targeting the frontal lobe, and one had a parietal operculum resection. In four cases, more than one surgical intervention was performed for each patient: two patients underwent temporal lobectomies following SAH, one underwent a Heschl’s gyrus resection following a leukotomy, and one had a total of four surgeries targeting the temporal lobe over a seven-year period. Pathology revealed hippocampal sclerosis in five patients (with patient #10 also having hippocampal focal cortical dysplasia), findings suggestive of mild malformation of cortical development in two patients, cystic ganglioglioma with associated dysplasia in one patient, and subpial gliosis in another patient. Pathology results were unremarkable for two other patients and unavailable in the three last patients.

### 3.3. Post-Surgery Failure Characteristics

Seizure recurrence ranged from the immediate post-operative period to one year: seven patients had seizure recurrence within one month, three after one month but before one year, and for the other four patients, the timing of seizure recurrence was unclear. Regarding semiology after initial surgical procedures, four patients reported no changes, four patients reported having the same semiology but with an increase in sleep-related seizures, and one patient reported having the same semiology but with increased intensity of hypermotor and vocalisation manifestations. Two patients reported no longer having BTC seizures, and another had a reduction in unaware episodes. Three patients reported new manifestations: patient #7 reported throat constrictive dysesthesias, patient #9 reported hemibody paresthesia, hypersalivation, occasional impaired awareness, and sleep-related hypermotor manifestations, and patient #10 reported auditory auras, hemicorporeal paresthesia, and occasional impaired awareness. Finally, one patient’s seizures lost their musicogenic/audiogenic quality following an anterior temporal lobectomy (patient #11).

MEG was performed in 13 patients and was the modality used to determine probable insular involvement in eight of these patients; the other five patients had insular involvement confirmed via other means. In fact, four patients underwent icEEG to confirm insular involvement, three of which also had insular involvement seen on MEG. Insular involvement was confirmed in two patients after they underwent insular resections that led to seizure freedom for more than two years.

Four patients had post-surgery-failure PET scans, with only one suggesting an insular focus (left temporo-insular hypometabolism). Two other patients had unremarkable/non-localizing PET scans, and the final patient had right mesial temporal hypometabolism. Ten patients underwent an ictal SPECT study post-surgery-failure. In five patients, ictal SPECTs localized activation to insular regions; the remaining ictal SPECTs showed activation in various temporal, frontal, and occipital regions.

Nine patients finally underwent re-operations that included an insular resection. Among these patients, seven achieved a favorable post-surgical seizure outcome (Engel classification I or II, see Table 3 for follow-up duration), while two did not. Among these two patients, one showed some improvement, and the other passed away from SUDEP in the months following surgery. Five patients declined to be re-operated.

Finally, no patients had pure insular epilepsy. Eight patients likely had temporal-plus epilepsy, whereas the remaining six likely had operculo-insular epilepsy.

## 4. Discussion

In this series, we reviewed cases of epilepsy surgery failure subsequently attributed to the contribution of the insular cortex to epilepsy genesis, with the hopes of improving the recognition of insular involvement in patients with refractory focal epilepsy.

Epilepsy surgery failure is relatively frequent, befalling 20–30% of temporal lobe surgeries and 30–50% of frontal lobe surgeries [1,2]. Approximately 4–14% of patients who have had an epilepsy surgery undergo a second operation [16].

Epilepsy surgery fails when epileptogenic tissues (and intact epileptogenic networks) remain post-operatively. This situation occurs if the epileptic focus is not fully resected or if a separate epileptic focus existed and was not identified in the presurgical evaluation. Buried deep in the Sylvian fissure, the insula generates seizures that tend to clinically resemble seizures generated in the temporal, frontal, and parietal lobes [5]. As a result, failure to identify the insula as playing a primary role in a patient’s epilepsy has been suggested to be a possible cause for epilepsy surgery failure [3]. In this small series of selected patients, we have indeed demonstrated that insular involvement, if not detected prior to epilepsy surgery, can be a cause of epilepsy surgery failure.

Identifying insular epilepsy can be challenging, but many clinical features are now recognized as clues that hint at insular involvement in epilepsy. Such clues include early paresthesia that are: (a) painful, (b) bilateral, (c) involving large cutaneous territories, or d) accompanied by a feeling of throat constriction. Early auditory, olfactory, and gustatory symptoms are also suggestive of insular seizures or involvement [5]. The insula is a multimodal sensory processing region, and a combination of different aura types (e.g., somatosensory, viscerosensory, olfacto-gustatory, etc.) should raise our suspicion for insular seizures [17]. Predominantly nocturnal hypermotor seizures occurring with a long latency period after arousal should also evoke a potential insular origin [18]. Other less specific clues include early viscerosensory and visceropsychic symptoms, as they are commonly encountered in medial temporal lobe seizures. Although rare, there have also been reports of patients with insular seizures that manifest as reflex seizures, gelastic seizures, and ecstatic seizures [5]. In this series, half of the patients had manifestations evoking possible insular involvement prior to their first epilepsy surgery. Among these patients, six of them had early somatosensory symptoms, six had a combination of auras, four had early auditory symptoms, and two had early olfacto-gustatory symptoms. Hence, a good understanding of the various semiologies of insular seizures is obviously important in the presurgical evaluation for epilepsy surgery but maybe not enough. On a separate note, all patients in this series, after their failed epilepsy surgery, presented either an unchanged or a similar seizure semiology, which further supported the notion of insular involvement.

Most non-invasive paraclinical investigations are limited in exploring insular involvement in epilepsy. Scalp EEG, both interictal and ictal, typically exhibit non-localizing abnormalities over the frontal, temporal, or central lead derivations [19]. The majority of patients in this series had scalp EEGs consistent with a temporal focus. Brain MRI can identify insular epileptogenic lesions, greatly supporting the diagnosis of insular epilepsy; however, it is not uncommon that brain MRI in patients with insular epilepsy yield normal or nonspecific findings [5]. In this series, only two patients had insular abnormalities on MRI. SPECT is thought to be of moderate value in identifying insular epilepsy [20,21]. Similarly, PET has been thought to generate inconsistent findings, though some recent studies may suggest otherwise [20,22,23]. In this series, in the pre-surgery-failure evaluation, only one SPECT study localized activation to the operculo-insular region, whereas all other SPECT studies were either non-localizing or localized to various other regions. Post-surgery-failure SPECT studies, however, localized activation to insular regions in half of the patients. As for PET scans, none of the pre-surgery-failure studies suggested an insular focus, and only one out of four post-surgery-failure studies suggested an insular focus. Our findings are therefore in support of the notion that SPECT and PET may be limited in detecting insular involvement in epilepsy, often yielding non-localizing or inconsistent results.

Among non-invasive modalities for investigating insular involvement in epilepsy, a mounting body of evidence points towards MEG as being the most promising [8,9,10,11,12,24,25,26]. MEG has been extensively studied in the presurgical evaluation of epilepsy, and surgical resection of MEG dipoles (including in the insula) has been associated with improved patient outcomes [27,28]. The current body of evidence suggests that MEG could be more effective than both SPECT and PET in identifying operculo-insular epilepsy [24]. Our series may add to our understanding of the utility of MEG in the evaluation of insular epilepsy, notably after an initial surgery failure. Only four MEGs were performed pre-surgically, with one showing a cluster of sources over the anterior insula. This low number of MEG studies prior to initial surgery was due to our limited understanding and access to MEG in the past; in fact, MEG was only available in our institutions from 2006 onwards and was only initially used for research purposes. MEGs were performed in thirteen of the fourteen patients after surgery failure, demonstrating insular involvement in twelve patients and used as a touchstone to determine insular involvement in eight of these patients. MEG was the most frequently used modality to determine insular involvement after surgery failure and often helped guide the decision for reoperation. However, since MEG results were used in this series as a criterion for confirming insular involvement, we cannot offer additional comments on the reliability of MEG for detecting insular epileptic activity post-surgery-failure. It has been proposed nevertheless that MEG may carry advantages for localisation in patients with previous surgeries since the signals are less altered by skull defects or surgical cavities as compared to other modalities [13].

Invasive icEEG (with insular sampling) remains the gold standard in diagnosing an insular focus. The insula is usually explored using depth electrodes, most often in stereoelectroencephalography (SEEG) [29]. In this series, only three patients had insular coverage during the presurgical evaluation preceding epilepsy surgery failure, albeit in a suboptimal way by current standards. These intracranial EEG studies were performed more than a decade ago when experience with insular epilepsy was still limited. At our institutions, the use of a combination of subdural grid/strip electrodes and depth electrodes for intracranial EEG through open craniotomy has largely been replaced by SEEG, and insular sampling has become more precise with multiple (orthogonal and oblique) insular electrodes dedicated to different insular subregions [29]. If these three patients had had better insular coverage, insular seizure onset zones could have been detected. Likewise, if the other eleven patients had benefited from insular depth electrodes, insular involvement could have been detected, and epilepsy surgery failure avoided.

Searching for an insular focus after an initial epilepsy surgery failure can be rewarding, as it may lead to a re-operation which may significantly improve a patient’s condition. Of the nine patients in our cohort who underwent re-operations which included insulectomies, seven achieved successful outcomes (i.e., Engel classes I or II). As such, careful presurgical and postsurgical evaluations geared at uncovering insular involvement appear necessary for optimizing patient outcomes. As for the surgical technique used, though open surgery of the insula remains common, minimally invasive techniques have been attempted. These techniques include, but are not limited to, laser interstitial thermal therapy (LITT), an appealing option that has been suggested to minimize damage to opercula and M2 vessels/perforators. Though none of the patients in this series underwent LITT, there is growing evidence that this technique may yield good outcomes. Our group recently published a review of minimally invasive techniques and found that 58% of patients having undergone LITT in the available literature (a total of 38 patients) had Engel class I outcomes [30].

It must be acknowledged that pure insular epilepsy is relatively rare. Most often, patients present seizure onset zones centered on the insula that extend to the opercula (i.e., insulo-opercular epilepsy) or centered on the temporal lobe that extend to the insula (i.e., temporal-plus epilepsy) [5,31]. In this series, no cases of pure insular epilepsy were reported; instead, eight cases were most likely temporal-plus epilepsies, whereas the remaining six cases were most likely operculo-insular epilepsies. Temporal-plus epilepsy has been reported as a major predictor of TLE surgery failure, but distinguishing this entity from pure TLE can be challenging [31]. Up until recently, icEEG was considered the only method to accurately make this distinction; on a practical level and in the presence of hippocampal sclerosis, one often proceeds with a direct anterior temporal resection.

Certain red flags are often not sufficient to deter from direct surgery. For example, occurrence during sleep, bradycardia, and somatosensory or olfactory-gustatory auras (from rapid spread to the insula) have been reported with anterior temporal lobe epilepsy, and extended temporo-insular hypometabolism is common in anterior temporal lobe epilepsy. In our series, none of the patients with MTS and with a presentation suggestive of a temporal focus underwent icEEG before their first surgery. Interestingly, recent literature has shed some light on non-invasive tests that may orient suspicion towards temporal-plus epilepsy rather than pure TLE. Chassoux et al. analyzed PET findings in a cohort of patients with MTS that underwent anteromesial temporal resection, comparing those with Engel IA outcomes to those with non-IA outcomes. The authors found that extra-temporal metabolic changes, including certain patterns of fronto-insular hypometabolism, correlated with poorer surgical outcomes (i.e., non-IA outcomes) [32]. Martire et al. analyzed the connectomic profiles of children with temporal-plus epilepsy and pure TLE using MEG and were able to develop a model that could robustly differentiate the two clinical entities [33].

This study featured certain limitations. Firstly, this study is limited by its sample size: only 14 cases of insular involvement after surgery failure were identified. A larger multi-centered study would be necessary to further explore the topic. Secondly, the manner by which we identified cases of epilepsy surgery failure attributed to insular involvement was not systematic. Though we aimed to only describe the cases of epilepsy surgery failure subsequently attributed to an insular focus in this series, a more ambitious study could have systematically gathered all cases of epilepsy surgery failure and offered comparisons between various subgroups. Thirdly, MEG was used as a criterion to confirm probable insular involvement in epilepsy. We acknowledge however that source localization of interictal spikes to the insula does not guarantee seizures are from the same area. Previous work from our group and others suggests nonetheless that MEG is useful in the identification of insular epilepsy [8]. Fourthly, this series is subject to inherent biases due to its retrospective nature. Finally, this series reflects the practice in tertiary care centers, presenting patients from a particularly refractory cohort, and may be less generalizable to other settings.

## 5. Conclusions

The present study is a series showcasing clinical features, paraclinical investigations, and surgery data of patients with epilepsy surgery failures who were subsequently found to have insular involvement, either by intracranial EEG, MEG, or demonstration of seizure freedom post-insulectomy. We have shown that insular involvement is a missed cause of epilepsy surgery failure. Ideally, a careful presurgical evaluation with attention to semiological aspects hinting at insular epilepsy should always be carried out. A high index of suspicion should be given to insular involvement particularly after epilepsy surgery failure and if semiology is suggestive of such an involvement. Our findings may support MEG as being a promising non-invasive method for investigating insular epileptic activity after an initial epilepsy surgery failure, though icEEG with insular sampling remains the gold standard. Uncovering insular involvement may allow for re-operations. Lowering our threshold for suspecting insular epilepsy may be necessary to help diminish epilepsy surgery failures.

## Figures and Tables

**Table 1:** Cohort summary.

	Number of Patients, *n* (%)
Male	11 (79)
Risk factors	
Febrile seizures	1 (7)
Family history	1 (7)
Meningoencephalitis	1 (7)
Perinatal complications	1 (7)
No pertinent risk factors	10 (71)
Semiology	
Focal-to-bilateral tonic-clonic seizures	11 (79)
Sleep-related seizures	10 (71)
Somatosensory symptoms	6 (43)
Oro-alimentary automatisms	6 (43)
Epigastric sensations	5 (36)
Hyperkinetic seizures	4 (29)
Clonic motor manifestations	4 (29)
Auditory illusions	4 (29)
Dystonic posturing	3 (21)
Olfactogustatory symptoms	3 (21)
Déjà-vu	3 (21)
Autonomic activation	3 (21)
Emotional manifestations	3 (21)
Hypersalivation	2 (14)
Language symptoms	2 (14)
Behavioral arrest	2 (14)
Throat constriction/dysesthesias	2 (14)
Upper extremity automatisms	2 (14)
Gelastic seizures	1 (7)
Scalp EEG	
Before failed surgery	14 (100)
Interictal	
Temporal	9 (64)
Frontal	3 (21)
Fronto-temporal	2 (14)
Ictal	
Temporal	7 (50)
Frontal	1 (7)
Fronto-temporal	1 (7)
Non-localizing	5 (36)
After failed surgery	13 (93)
Interictal	
Temporal	8 (62)
Frontal	0 (0)
Fronto-temporal	4 (31)
Non-localizing	1 (7)
Ictal	
Temporal	9 (69)
Frontal	0 (0)
Fronto-temporal	2 (15)
Non-localizing	2 (15)
MRI	
Hippocampal sclerosis	5 (36)
Temporal lobe atrophy without hippocampal sclerosis	2 (14)
Insular abnormalities	2 (14)
Unremarkable (non-lesional)	3 (21)
No preoperative MRI available	2 (14)
SPECT	
Before failed surgery	9 (64)
Insular activation	0 (0)
No insular activation	8 (89)
Non-localizing	1 (11)
After failed surgery	10 (71)
Insular activation	5 (50)
No insular activation	5 (50)
PET	
Before failed surgery	8 (57)
Extra-insular localization	8 (100)
After failed surgery	4 (29)
Insular localization	1 (25)
Extra-insular localization	1 (25)
Non-localizing	2 (50)
MEG	
Before failed surgery	4 (29)
Insular-plus localization	1 (25)
Extra-insular localization	3 (75)
After failed surgery	13 (93)
Insular-plus localization	12 (92)
Extra-insular localization	1 (8)
Intracranial EEG	
Before failed surgery	8 (57)
Insula sampling	3 (38)
After failed surgery	5 (36)
Failed surgery	
Temporal	8 (57)
Extra temporal (excluding insula)	6 (43)
Re-operated with insulectomy	9 (64)
Outcomes after last operation (n = 9)	
Engel IA	2 (22)
Engel IIB	4 (44)
Engel IID	1 (11)
Engel IIIA	1 (11)
Engel IVC	1 (11)

EEG = electroencephalography; MEG = magnetoencephalography; MRI = magnetic resonance imaging; PET = positron emission tomography; SPECT = single-photon emission computed tomography.

**Table 2:** Baseline, pre-surgical, and peri-surgical characteristics of included patients.

Patient ID	Gender	Epilepsy Risk Factors	Age at Onset (Years)	Semiology	Sleep-Related (Y/N)	Scalp EEG	icEEG	MRI	PET	SPECT	MEG	Surgeries Performed	Pathology
1	M	-	20	Focal with impaired awareness	Y	Interictal: R T Ictal: R T	-	R HS	↓R MES-T (post-SAH)	R ANT T (post-SAH)	R ANT T, orbito-F, and aINS (post-SAH)	1. R SAH 2. R T LBC	1. HS and dentate disorganization 2. -
				Olfactogustatory aura Epigastric rising, nausea, hypersalivation, ±R blinking, R LE paresthesia, oroalimentary automatisms ±Déjà vu Sleep-related complex motor manifestations									
2	F	-	9	Focal with impaired awareness ± BTC	Y	Interictal: R F Ictal: NL	R SUP F R INF F/OPC (insula sampled)	UN	↓R F	1. R F OPC 2. R orbito-F and R ANT MES-F	-	R polar and MES-F partial LBC	Subpial gliosis, no dysplasia
				No aura Arousal and pedaling									
3	M	-	6	Focal with impaired awareness ± to BTC	Y	Interictal: R F Ictal: NL	R POST-MED, INF, and polar F, spread to ANT cingulate (insula sampled)	Possible discrete R HA	↑R LAT T (post-ictal)	1. L basal ganglia 2. MES-F, ANT cingulate lateralization unclear 3 and 4. NL	L MES-F	R F-polar CTC and ANT cingulate gyrus	Mild MCD (type II)
				Aura—R ear paresthesia and auditory L eye and head deviation, grimace, R arm dystonia, R LE pedaling Ending with gelastic seizure or clonic L hemibody followed by BTC									
4	M	-	11	Focal with impaired awareness ± BTC	N	Interictal: R F T Ictal: NL	NL	UN	-	-	-	R F CTC	-
				Heat, dizzy, behavioral arrest, ±R then L head and L UE clonic ±post ictal aphasia									
5	M	FHx+ with 2 uncles on maternal side	40	Focal with impaired awareness ± BTC	Y	Interictal: R T Ictal: R T	-	R MTS	↓R MES-T	NL	-	R SAH	MTS
				Déjà-vu, oroalimentary automatisms, UE dystonic posturing									
6	M	-	35	Focal with impaired awareness, rare BTC	Y	Interictal: L F Ictal: Bi-F	R MES-F and F-polar (insula not sampled)	1. Mild dilation of R T horn, R POST T minor atrophy 2. Minor L HA	1. ↓bi-LAT MES-T (R > L) 2. ↓R F-polar and R MES-T	1 and 2. NL 3. R INF F gyrus and R F-polar	R aINS, orbito-F cortex, frontal OPC	R MES-F CTC	Gliosis, mild MCD (microdysgenesis)
				Epigastric rising, facial flushing, and hand automatisms									
7	M	-	15	Focal with impaired awareness ± BTC	Y	Interictal: L F T Ictal: L F T	L F	-	-	-	-	L ANT-F LBC	UN
				R hand clonic, R UE dystonic posturing, and oroalimentary automatisms Post ictal aphasia									
8	F	-	14	Focal aware	N	Interictal: Bi-T Ictal: NL	L HC, L Heschl’s (insula sampling attempted but failed)	L pINS and Heschl’s gyrus junction dysplasia	-	-	-	1. Leukotomy 2. L Heschl’s gyrus partial resection	Cystic ganglioglioma with dysplasia
				Auditory aura with descending dysesthesias from thorax to pelvis									
9	M	Atypical febrile seizure at 6 months	28	Focal aware ± BTC	N	Interictal: R T Ictal: R T	-	HS	↓R MES and LAT T	Ictal not performed Interictal: R orbito-F	-	1. R SAH 2. R T LBC	HS
				Epigastric rising, olfactogustatory aura, sadness									
10	M	-	10	Focal with impaired awareness ± rare BTC	U	Interictal: R T Ictal: R T	-	R HS	↓R MES-T	1. Discrete R T-polar 2. Discrete R T		R ANT T LBC	HS with FCD
11	M	Meningoencephalitis-litis during youth	4	Focal with impaired awareness ± BTC	Y	Interictal: R T Ictal: R T	-	-	-	-	-	R ANT T LBC (1998)	-
				Musicogenic with L head version, oroalimentary automatisms, L arm clonic movements									
12	M	-	16	Focal aware	Y	Interictal: L T Ictal: NL	L POST-T (insula not sampled)	Discrete thickening of L INS L ANT-MES T arachnoid cyst	-	-	-	Small L operculo-P resection	UN
				Sharp pain in R side of head, auditory Sleep-related complex motor manifestations									
13	F	Perinatal complications	2	Focal with impaired awareness	Y	Interictal: L T Ictal: L T	-	L HS + HC and para-HC atrophy	↓L MES-T extending to T-polar and LAT T	L LAT and SUP T L middle F gyrus	L MES-T	L ANT T LBC	HS
				Aura déjà-vu, depersonalization, bi-LAT paresthesia, epigastric rising, nausea, olfactogustatory, oroalimentary automatisms, autonomic (sleep-related BTC seizures only in childhood)									
14	M	-	9	Focal with impaired awareness ± BTC	Y	Interictal: R T Ictal: R T	1. R AG, HC, POST T 2. R LAT T neocortex (insula not sampled)	UN	-	Ictal not performed Interictal: R T	-	4 surgeries over 7 years involving R T lobe	-
				Auditory aura Epigastric rising, throat dysesthesia, hypersalivation, paresthesia in the L UE ± L face and LE, ±AOC with elevation of L UE and L head deviation Sleep-related oroalimentary automatisms									

ANT = anterior AOC = alteration of consciousness; BTC = bilateral tonic-clonic; CTC = cortectomy; CTL = contralateral; EEG = electroencephalography (ic- = intracranial-, v- = video-); FCD = focal cortical dysplasia; FHx = family history; HC = hippocampus; HA = hippocampal atrophy; HS = hippocampal sclerosis; HHE = hemiconvulsion hemiplegia epilepsy; INF = inferior; INS = insula (aINS = anterior insula, pINS = posterior insula); L = left; LAT = lateral; LE = lower extremity; LBC = lobectomy; MCD = malformation of cortical development; MED = medial; MEG = magnetoencephalography; MES = mesial; MRI = magnetic resonance imaging; N = no; NL = non-localizing; O = occipital; OPC = operculum; P = parietal; PET = positron emission tomography; POST = posterior; R = right; SAH = selective amygdalohippocampectomy; SPECT = single-photon emission computed tomography; SUP = superior T = temporal; TBI = traumatic brain injury; UE = upper extremity; UN = unremarkable; Y = yes.

**Table 3:** Post-surgery failure characteristics of included patients.

Patient ID	Recurrence Time	Semiology	Scalp EEG	icEEG	MRI	PET	SPECT	MEG	EEG-fMRI	Surgery Targeting Insula	Outcome, Medication, and Engel	Probable Epilepsy Type
1	2 weeks	Unchanged	Interictal: R T Ictal: R T	R INS *	R T-O gadolinium enhancing lesion	-	R F-T-P	R aINS, orbito-F	R INS (weak activation)	2011: R INSC	Seizure-free, no ASM PO exacerbation of OCD symptoms Engel IA (at 3-year F/U)	T+
2	6 to 12 months	As previously with increased intensity of pedaling (R > L) and vocalizations	Interictal: R F T Ictal: R T	-	-	-	1. Possible R F 2. R T-O junction	1. R aINS and pINS 2. R pINS and T OPC, middle and INF F *	R F OPC and INS	2014: R F CTC and R INSC	Decreased seizure intensity and frequency 3 ASMs VNS installed in 2019 Engel IIIA (at 6-year follow-up)	OI
3	Days to weeks (small reduction in frequency)	Unchanged	Interictal: R F T Ictal: R F T	Peri-operative F and insular sampling inconclusive	Possible thickening of R INF F gyrus (other MRIs UN)	-	R F	R POST middle and INF F 2 MEG post-INSC 1. R INS, orbito-F, POST-LAT and POST F 2. POST third of Sylvian fissure	-	2012: R INF F gyrus resection and superior INSC	Seizure-free for 2 years; seizure recurrence, well-controlled using 3 ASMs for additional 2 years; seizure-free for next 4 years with the same 3 ASMs Engel IIB * (at 8-year F/U)	OI
4	Unclear	Unchanged with no recurrent BTC seizures	Interictal: R F T Ictal: NL	-	PO changes only	-	1. Possible R F 2: R T-O	aINS, T, orbito-F *	-	-	At last F/U, seizures well-controlled using 4 ASMs	T+
5	Unclear (small reduction in frequency)	Sleep-related only	Interictal: R T Ictal: R T	-	T2/Flair hyper-intensity in POST HC and para-HC, ANT HC resected	↓R MES-T	-	1. UN 2. Inconclusive 3. ANT T and aINS *	-	2014: R T LBC and INSC	Recurrence 1 month PO Few sleep-related seizures On 2 ASMs New epilepsy-related cognitive impairment Engel IID (at 4-year F/U)	T+
6	4 months	More often sleep-related	-	-	PO changes only	-	Pre-F and ANT F INS OPC	R ANT F-INS OPC *	-	2013: R orbito-F and F operculo-insular corticectomy	Only 1 nocturnal seizure during ASM dose change during 4 years of F/U On 2 ASMs Engel IIB (at 4-year F/U)	OI
7	Immediate	As previously with added throat dysesthesia and new diurnal seizures	Interictal: L F T Ictal: L F T	-	PO changes only	-	L head of caudate with extension to orbito-F, INS, and MES-T	L F OPC, orbito-F, and aINS *	-	-	Adequate control on 3 ASMs (only sleep-related)	OI
8	Immediate (small reduction in frequency)	Unchanged	Interictal: Bi-T Ictal: NL	-	Persistent micro-cystic lesion in L INS	↓L INS-T	L > R INS/basal ganglia	R INS-MES-T and rare L INS *	-	-	-	T+
9	1 month	As previously with L hemi-body paresthesia, hypersalivation ±AOC ±sleep-related hypermotor	Interictal: R T Ictal: R T	pINS with spread to ANT orbito-F and peri-sylvian *	PO changes, abnormal T2 signal extending into R INS	-	-	R aINS, posterior Sylvian fissure	-	2013: R INSC	Sudden unexpected death in epilepsy 3 months PO (residual orbito-F focus) Engel IVC (at 3-month F/U)	T+
10	1 aura at 1 month, full seizure recurrence at 1 year	L LE or full body paresthesia and auditory aura, ±AOC	Interictal: R T Ictal: R T	Declined by patient	PO changes only	-	-	R residual SUP T gyrus and INS *	-	-	Seizure-free since introduction of levetiracetam at 4-year F/U (on 3 ASMs at 7-year F/U)	T+
11	Unclear	More often sleep-related; no musico-genic aura, no recurrent BTC	Interictal: R T Ictal: R T	-	PO changes only	-	-	R aINS, orbito-F *	-	-	-	OI
12	Unclear	Unchanged though with more sleep-related	Interictal: L T Ictal: L T	L pINS and operculo-P *	PO changes, cystic lesion in L ANT T	NL	1. R INS 2. UN	L POST T and pINS 1 dipole in aINS	-	2012: L operculo-INSC	Seizure-free on 2 ASMs Engel IA (at 1-year F/U)	OI
13	5 months	Unchanged	Interictal: NL Ictal: L T	L INF INS and SUP T POST to resection (stimulation of INF INS provoked déjà-vu) *	PO changes with possible residual grey matter from HC/ /AG	UN	INF T-O	L MES-T	-	2018: L radical T LBC and INSC	Recurrent auras, though less frequent Rare seizures with AOC On 2 ASMs Engel IIB (at 2-year F/U)	T+
14	<1 month	Unchanged with rarer AOC	Interictal: R T Ictal: R T	-	PO changes only	-	R aINS	-	2004: R aINS	2005: R supramarginal gyrus and partial aINS (limen)	Improved seizure control on 3 ASMs Mostly auras, rare seizures (2–5 per year) Engel IIB * (at 15-year F/U)	T+

AG = amygdala; ANT = anterior AOC = alteration of consciousness; ASM = antiseizure medication; BTC = bilateral tonic-clonic; CTC = cortectomy; EEG = electroencephalography (ic- = intracranial-, v- = video-); F/U = follow-up; HC = hippocampus; INF = inferior; INS = insula (aINS = anterior insula, pINS = posterior insula); INSC = insulectomy; L = left; LAT = lateral; LE = lower extremity; LBC = lobectomy; MED = medial; MEG = magnetoencephalography; MES = mesial; MRI = magnetic resonance imaging (f- = functional); NL = non-localizing; O = occipital; OCD = obsessive-compulsive disorder; OI = operculo-insular; OPC = operculum; P = parietal; PET = positron emission tomography; PO = post-operative; POST = posterior; R = right; SPECT = Single-photon emission computed tomography; SUP = superior; T = temporal; T+ = temporal-plus; UN = unremarkable; VNS = vagus nerve stimulator. * Manner by which insular involvement was determined (icEEG, MEG, or response to insulectomy).

## Data Availability

Data not provided in the article due to space limitations may be shared (anonymized) at the request of any qualified investigator.

## References

[1] McIntosh A.M., Kalnins R.M., Mitchell L.A., Fabinyi G.C., Briellmann R.S., Berkovic S.F. (2004). Temporal lobectomy: Long-term seizure outcome, late recurrence and risks for seizure recurrence. Brain J. Neurol..

[2] Jeha L.E., Najm I., Bingaman W., Dinner D., Widdess-Walsh P., Luders H. (2007). Surgical outcome and prognostic factors of frontal lobe epilepsy surgery. Brain J. Neurol..

[3] Harroud A., Bouthillier A., Weil A.G., Nguyen D.K. (2012). Temporal lobe epilepsy surgery failures: A review. Epilepsy Res. Treat..

[4] Ryvlin P., Kahane P. (2005). The hidden causes of surgery-resistant temporal lobe epilepsy: Extratemporal or temporal plus?. Curr. Opin. Neurol..

[5] Obaid S., Zerouali Y., Nguyen D.K. (2017). Insular Epilepsy: Semiology and Noninvasive Investigations. J. Clin. Neurophysiol..

[6] Gras-Combe G., Minotti L., Hoffmann D., Krainik A., Kahane P., Chabardes S. (2016). Surgery for Nontumoral Insular Epilepsy Explored by Stereoelectroencephalography. Neurosurgery.

[7] Kakisaka Y., Alkawadri R., Wang Z.I., Enatsu R., Mosher J.C., Dubarry A.S., Alexopoulos A.V., Burgess R.C. (2013). Sensitivity of scalp 10–20 EEG and magnetoencephalography. Epileptic Disord. Int. Epilepsy J. Videotape.

[8] Mohamed I.S., Toffa D.H., Robert M., Cossette P., Berube A.A., Saint-Hilaire J.M., Bouthillier A., Nguyen D.K. (2020). Utility of magnetic source imaging in nonlesional focal epilepsy: A prospective study. Neurosurg. Focus.

[9] Chourasia N., Quach M., Gavvala J. (2021). Insular Magnetoencephalography Dipole Clusters in Patients With Refractory Focal Epilepsy. J. Clin. Neurophysiol..

[10] Heers M., Rampp S., Stefan H., Urbach H., Elger C.E., von Lehe M., Wellmer J. (2012). MEG-based identification of the epileptogenic zone in occult peri-insular epilepsy. Seizure.

[11] Park H.M., Nakasato N., Tominaga T. (2012). Localization of abnormal discharges causing insular epilepsy by magnetoencephalography. Tohoku J. Exp. Med..

[12] Bagic A.I., Funke M.E., Kirsch H.E., Tenney J.R., Zillgitt A.J., Burgess R.C. (2020). The 10 Common Evidence-Supported Indications for MEG in Epilepsy Surgery: An Illustrated Compendium. J. Clin. Neurophysiol..

[13] Okada Y.C., Lahteenmaki A., Xu C. (1999). Experimental analysis of distortion of magnetoencephalography signals by the skull. Clin. Neurophysiol. Off. J. Int. Fed. Clin. Neurophysiol..

[14] Isnard J., Guenot M., Ostrowsky K., Sindou M., Mauguiere F. (2000). The role of the insular cortex in temporal lobe epilepsy. Ann. Neurol..

[15] Surbeck W., Bouthillier A., Weil A.G., Crevier L., Carmant L., Lortie A., Major P., Nguyen D.K. (2011). The combination of subdural and depth electrodes for intracranial EEG investigation of suspected insular (perisylvian) epilepsy. Epilepsia.

[16] Surges R., Elger C.E. (2013). Reoperation after failed resective epilepsy surgery. Seizure.

[17] Laoprasert P., Ojemann J.G., Handler M.H. (2017). Insular epilepsy surgery. Epilepsia.

[18] Proserpio P., Cossu M., Francione S., Tassi L., Mai R., Didato G., Castana L., Cardinale F., Sartori I., Gozzo F. (2011). Insular-opercular seizures manifesting with sleep-related paroxysmal motor behaviors: A stereo-EEG study. Epilepsia.

[19] Levy A., Yen Tran T.P., Boucher O., Bouthillier A., Nguyen D.K. (2017). Operculo-Insular Epilepsy: Scalp and Intracranial Electroencephalographic Findings. J. Clin. Neurophysiol..

[20] Fei P., Soucy J.P., Obaid S., Boucher O., Bouthillier A., Nguyen D.K. (2018). The Value of Regional Cerebral Blood Flow SPECT and FDG PET in Operculoinsular Epilepsy. Clin. Nucl. Med..

[21] Sala-Padro J., Fong M., Rahman Z., Bartley M., Gill D., Dexter M., Bleasel A., Wong C. (2019). A study of perfusion changes with Insula Epilepsy using SPECT. Seizure.

[22] Zhao B., Seguin C., Ai L., Sun T., Hu W., Zhang C., Wang X., Liu C., Wang Y., Mo J. (2020). Aberrant Metabolic Patterns Networks in Insular Epilepsy. Front. Neurol..

[23] Wang X., Hu W., McGonigal A., Zhang C., Sang L., Zhao B., Sun T., Wang F., Zhang J.G., Shao X. (2019). Electroclinical features of insulo-opercular epilepsy: An SEEG and PET study. Ann. Clin. Transl. Neurol..

[24] Mohamed I.S., Gibbs S.A., Robert M., Bouthillier A., Leroux J.M., Khoa Nguyen D. (2013). The utility of magnetoencephalography in the presurgical evaluation of refractory insular epilepsy. Epilepsia.

[25] Yin C., Zhang X., Chen Z., Li X., Wu S., Lv P., Wang Y. (2019). Detection and localization of interictal ripples with magnetoencephalography in the presurgical evaluation of drug-resistant insular epilepsy. Brain Res..

[26] Ma K., Luan G., Wang X., Luo S., Qin L., Teng P., Guan Y., Zhao M., Wang J., Wang M. (2021). Magnetoencephalography STOUT Method Adapted to Radiofrequency Thermocoagulation for MR-Negative Insular Epilepsy: A Case Report. Front. Neurol..

[27] Almubarak S., Alexopoulos A., Von-Podewils F., Wang Z.I., Kakisaka Y., Mosher J.C., Bulacio J., Gonzalez-Martinez J., Bingaman W., Burgess R.C. (2014). The correlation of magnetoencephalography to intracranial EEG in localizing the epileptogenic zone: A study of the surgical resection outcome. Epilepsy Res..

[28] Fischer M.J., Scheler G., Stefan H. (2005). Utilization of magnetoencephalography results to obtain favourable outcomes in epilepsy surgery. Brain J. Neurol..

[29] Ryvlin P., Picard F. (2017). Invasive Investigation of Insular Cortex Epilepsy. J. Clin. Neurophysiol..

[30] Ryvlin P., Nguyen D.K. (2021). Insular seizures and epilepsies: Ictal semiology and minimal invasive surgery. Curr. Opin. Neurol..

[31] Barba C., Minotti L., Job A.S., Kahane P. (2017). The Insula in Temporal Plus Epilepsy. J. Clin. Neurophysiol..

[32] Chassoux F., Artiges E., Semah F., Laurent A., Landré E., Turak B., Gervais P., Helal B.O., Devaux B. (2017). (18)F-FDG-PET patterns of surgical success and failure in mesial temporal lobe epilepsy. Neurology.

[33] Martire D.J., Wong S., Workewych A., Pang E., Boutros S., Smith M.L., Ochi A., Otsubo H., Sharma R., Widjaja E. (2020). Temporal-plus epilepsy in children: A connectomic analysis in magnetoencephalography. Epilepsia.

